# A Rare Case of B-cell Prolymphocytic Leukemia: The First Documented Diagnosis in Georgia With a Comprehensive Literature Review

**DOI:** 10.7759/cureus.76787

**Published:** 2025-01-02

**Authors:** Giorgi Maisuradze, Giorgi Akhvlediani, Elene Dzodzuashvili, Tamar Gersamia, Maia Sturua

**Affiliations:** 1 Biomedical Sciences, Georgian-American University, Tbilisi, GEO; 2 Internal Medicine, Ilia State University, Tbilisi, GEO; 3 Pulmonary and Critical Care Medicine, Tbilisi State Medical University, Tbilisi, GEO; 4 Endocrinology, American Hospital Tbilisi, Tbilisi, GEO; 5 Hematology and Oncology, Jerarsi Clinic, Tbilisi, GEO; 6 Morphology, Molecular Medicine Research Center - Primax, Tbilisi, GEO

**Keywords:** b-cell prolymphocytic leukemia (b-pll), georgia, minimal residual disease by flow cytometry, rare malignancy, rituximab-bendamustine

## Abstract

B-cell prolymphocytic leukemia (B-PLL) is a rare and aggressive malignancy of mature B-cells associated with poor prognosis, limited treatment options, and a median survival of approximately three years. We report the first documented case of B-PLL in the country of Georgia, involving a 64-year-old male presenting with fever, night sweats, weight loss, and generalized lymphadenopathy. Initial investigations revealed significant lymphocytosis and a predominance of prolymphocytes on the peripheral smear. The diagnosis was confirmed through bone marrow aspirate analysis and flow cytometry, demonstrating clonal B-cell proliferation with a characteristic immunophenotype. The patient was treated with a rituximab-bendamustine (R-B) regimen, achieving complete remission with no detectable minimal residual disease. Remarkably, he remains in clinical remission two years post-diagnosis, underscoring the potential of early and precise diagnosis combined with individualized therapeutic strategies in managing B-PLL. This case highlights the importance of further research to optimize treatment approaches and improve outcomes in this rare and challenging disease.

## Introduction

B-cell prolymphocytic leukemia (B-PLL) is an exceedingly rare and clinically aggressive hematologic malignancy, first delineated as a distinct entity in the late 20th century [1]. It accounts for fewer than 1% of all mature B-cell leukemias and lymphomas and predominantly affects older individuals, with a median age at diagnosis in the late 60s [1,2]. B-PLL is characterized by marked lymphocytosis and a predominance of prolymphocytes, which typically constitute more than 55% of circulating lymphocytes [2,3,4]. Clinical manifestations often include profound splenomegaly, anemia, thrombocytopenia, and systemic B symptoms such as fever, night sweats, unintentional weight loss, and fatigue. These symptoms, combined with rapidly rising white blood cell counts, often necessitate prompt intervention [4,5].

Due to its rarity, the incidence of B-PLL is not well-established, and its prognosis is notably poor, with median survival rarely exceeding three years [1,2,4]. Conventional treatments aim to alleviate symptoms and improve quality of life rather than achieve a cure, as standard regimens often yield partial or short-lived responses [3,4]. Commonly utilized regimens include fludarabine-based therapies and combinations such as fludarabine, cyclophosphamide, and rituximab (FCR) or rituximab-bendamustine (R-B) [6]. While these regimens may provide clinical benefits, durable remissions are infrequent. Novel therapies, including Bruton tyrosine kinase (BTK) inhibitors (e.g., ibrutinib and acalabrutinib) and BCL-2 inhibitors (e.g., venetoclax), have shown promise in case reports and small series [6-7]. Hematopoietic stem cell transplantation (HCT) remains the only potentially curative option for younger, fit patients, though it is associated with significant risks [5,6,7]. Despite these advancements, the lack of robust data leaves much of B-PLL management extrapolated from studies of chronic lymphocytic leukemia (CLL) [5,7].

This case report adds to the limited body of literature by presenting the first documented case of B-PLL in Georgia, highlighting unique challenges in diagnosis and treatment. Remarkably, the patient achieved exceptional long-term survival following an R-B regimen, demonstrating the potential for tailored therapeutic strategies to surpass traditional outcomes in this highly aggressive disease.

## Case presentation

A 64-year-old male presented with a three-month history of persistent fever, night sweats, significant unintentional weight loss, and generalized lymphadenopathy. On examination, multiple enlarged, non-tender lymph nodes were palpable in the cervical, axillary, and inguinal regions; however, there was no evidence of hepatosplenomegaly. The patient denied any recent infections or systemic illnesses. His past medical history was notable for arterial hypertension, a hemorrhagic stroke two years prior (resulting in residual left-sided weakness), and a remote history of successfully treated pulmonary tuberculosis. Initial laboratory investigations revealed markedly elevated white blood cell counts (213.82 × 10^9/L) and a strikingly high *monocyte* count (145.57 × 10^9/L). However, upon review of the peripheral blood smear, these cells were identified as prolymphocytes rather than true monocytes, indicating that the automated hematology analyzer had erroneously classified prolymphocytes in the monocyte population. Consequently, the actual monocyte count is significantly lower than reported by the machine (Table 1). 

**Table 1:** Initial complete blood count (CBC) results before the diagnosis of B-cell prolymphocytic leukemia. This table displays the patient’s CBC values obtained during the initial clinical evaluation, before the diagnosis of B-cell prolymphocytic leukemia, with corresponding reference ranges for comparison. Note: The automated hematology analyzer did not register any neutrophils in this sample, likely due to interference by the unusually high leukocyte count and overlapping cell populations. A manual smear review was, therefore, essential for an accurate differential assessment.

Parameter	Patient value	Reference range
White blood cell count (WBC)	213.82 × 10⁹/L	4.0-11.0 × 10⁹/L
Red blood cell count (RBC)	2.87 × 10¹²/L	4.5-5.9 × 10¹²/L
Hemoglobin (HGB)	6.1 g/dL	13.5-17.5 g/dL (male)
Hematocrit (HCT)	20%	40%-52%
Mean corpuscular volume (MCV)	94.1 fL	80-100 fL
Mean corpuscular hemoglobin (MCH)	28.2 pg	27-31 pg
Mean corpuscular hemoglobin concentration (MCHC)	30%	32%-36%
Red cell distribution width (RDW-CV)	15.7%	11.5%-14.5%
Platelets (PLT)	226 × 10⁹/L	150-450 × 10⁹/L
Mean platelet volume (MPV)	10.1 fL	7.5-11.5 fL
Lymphocyte count (LYMPH#)	76.54 × 10⁹/L	0.88-4.0 × 10⁹/L
Lymphocyte percentage (LYMPH%)	35.0%	20%-40%
Monocyte count (MONO#)	145.57 × 10⁹/L	0.08-1.0 × 10⁹/L
Monocyte percentage (MONO%)	68.1%	2%-10%
Eosinophil count (EO#)	0.18 × 10⁹/L	0.044-0.5 × 10⁹/L
Eosinophil percentage (EO%)	0.1%	1%-5%
Basophil count (BASO#)	0.06 × 10⁹/L	0-0.1 × 10⁹/L
Basophil percentage (BASO%)	0%	0-1%

**Figure 1:**
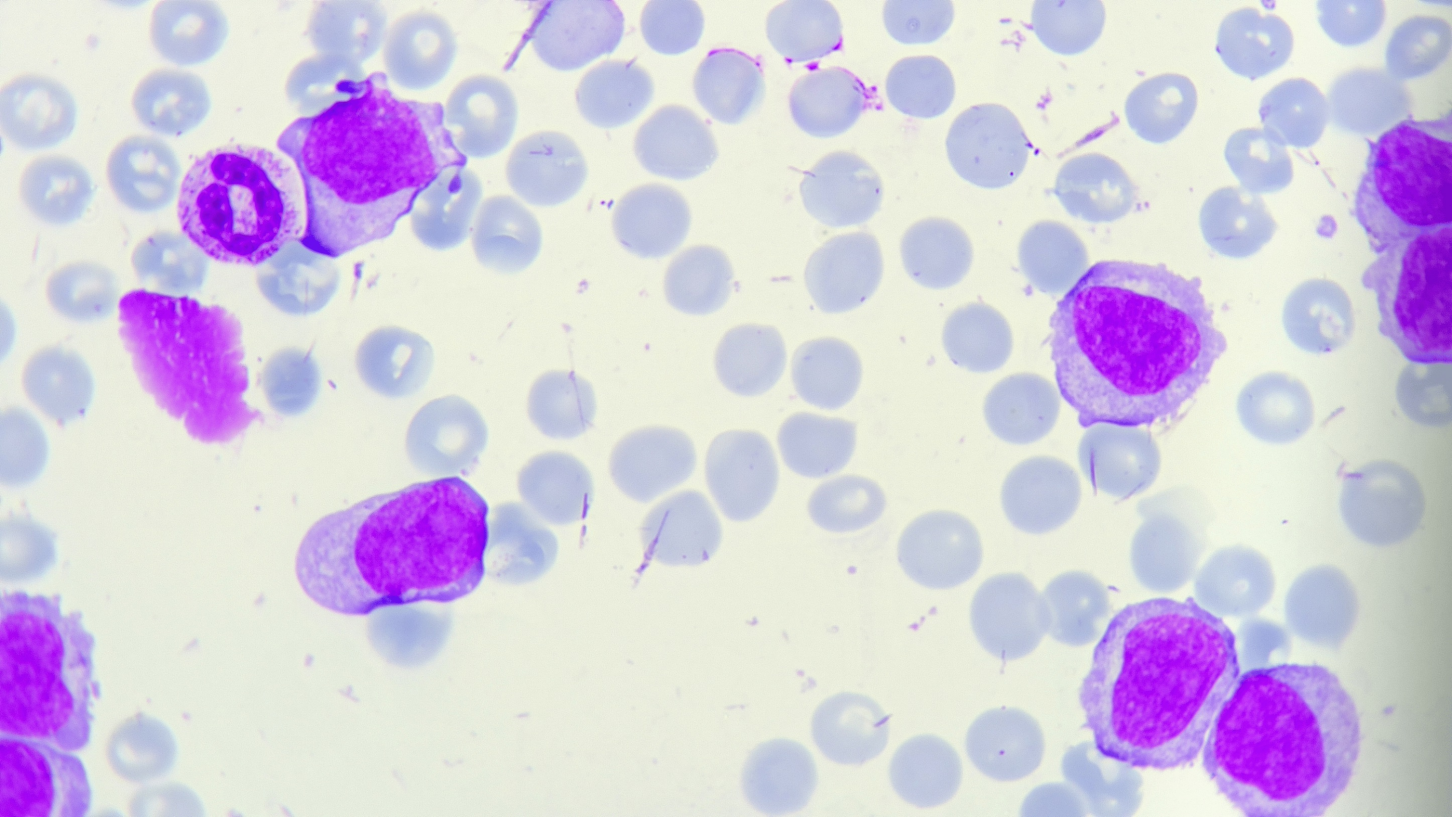
Peripheral blood smear from a patient with B-cell prolymphocytic leukemia (B-PLL). The image was acquired using a 10× ocular and a 100× oil-immersion objective lens, following Wright–Giemsa staining. Several large prolymphocytes with prominent nucleoli and scant basophilic cytoplasm are visible, exhibiting a high nuclear-to-cytoplasmic ratio. Red blood cells (RBCs) appear reduced in density, consistent with the patient’s anemia, and are distributed throughout the field.

Peripheral blood smear analysis revealed that more than 55% of the circulating leukocytes were prolymphocytes. These atypical cells displayed large, round to slightly oval nuclei with a high nuclear-to-cytoplasmic ratio, accompanied by well-defined, prominent nucleoli and scant, basophilic cytoplasm. The nuclear chromatin was moderately condensed - more so than what is typically observed in lymphoblasts yet still less dense than in fully mature lymphocytes - further underscoring the intermediate nature of these cells. Occasional smudge cells were also noted but did not comprise a significant proportion of the smear (Figure 1).

Taken together with the marked leukocytosis and the patient’s clinical presentation, these peripheral smear findings strongly suggested a highly aggressive lymphoid malignancy, prompting additional immunophenotypic and molecular investigations. The high percentage of prolymphocytes in the peripheral circulation is particularly characteristic of B-PLL, distinguishing it from other chronic lymphoproliferative disorders where fully mature lymphocytes or other cell types predominate.

**Figure 2:**
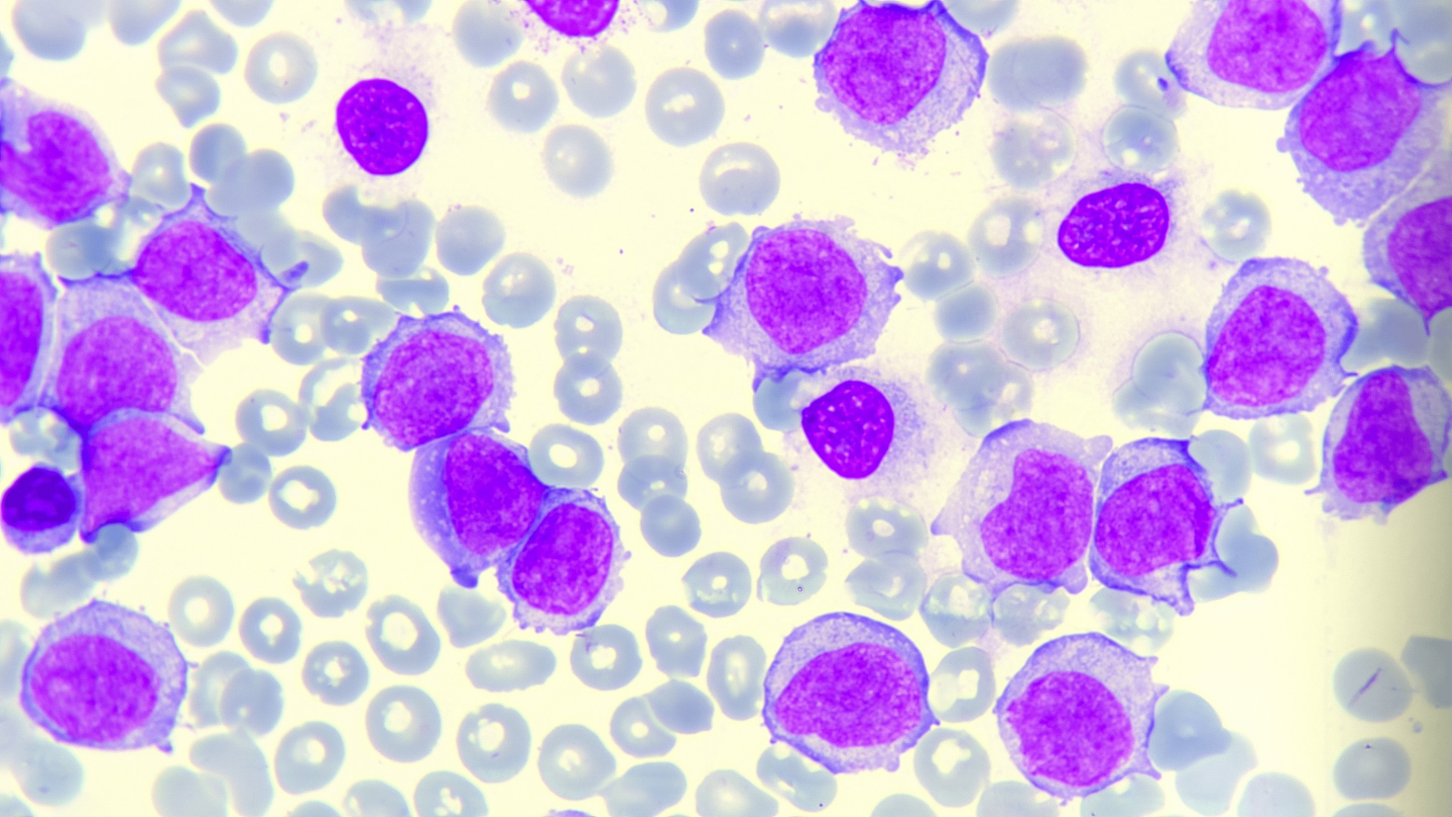
Bone marrow smear of a patient with B-cell prolymphocytic leukemia (B-PLL). The bone marrow smear from a patient with B-cell prolymphocytic leukemia (B-PLL) reveals significant infiltration with large prolymphocytes as the predominant cell type. These cells are characterized by large nuclei with a high nuclear-to-cytoplasmic ratio, moderately condensed chromatin, and prominent, centrally located nucleoli. The cytoplasm is pale basophilic, moderate in amount, and lacks visible granules. The background shows a sparse presence of erythroid and myeloid precursors, indicative of extensive bone marrow involvement by the leukemic process, with occasional platelet fragments visible. The smear was stained using the Wright-Giemsa staining technique, which highlights nuclear morphology and cytoplasmic features with excellent contrast. The image was observed under a microscope using a 10x ocular lens and a 100x oil immersion objective lens, providing a total magnification of 1000x. These findings are consistent with the diagnosis of B-PLL, a rare and aggressive B-cell malignancy.

Evaluation of the bone marrow aspirate and biopsy demonstrated a markedly hypercellular marrow. Morphologic assessment identified a preponderance of relatively immature lymphoid cells characterized by basophilic, plasmacytoid cytoplasm and a small nuclear vacuole, consistent with prolymphocytes (Figure 2). A myeloperoxidase (MPO) stain was negative, ruling out a myeloid origin. In light of these findings, flow cytometric analysis was deemed necessary to further delineate the immunophenotype and confirm the clonal nature of the abnormal B-cell population.

**Figure 3:**
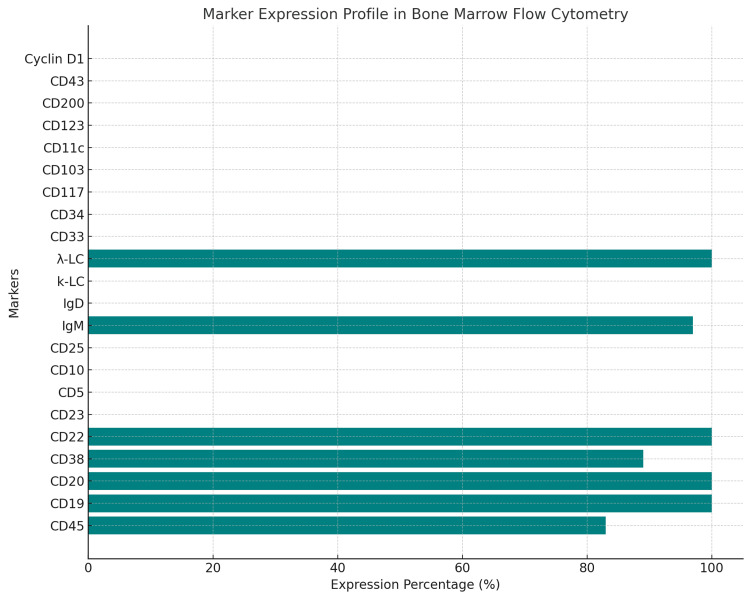
Marker expression profile in bone marrow flow cytometry. This horizontal bar chart illustrates the expression percentages of various immunophenotypic markers identified in bone marrow flow cytometric analysis. The analysis confirmed a clonal population of B cells characterized by high expression of CD19, CD20, CD22, and CD38, along with bright surface immunoglobulin (IgM, 97%) and λ light chain restriction (100%). Notably, markers typically associated with other B-cell neoplasms, including CD23, CD5, CD10, CD25, CD33, and CD34, were absent (0%). Similarly, markers indicative of myeloid or hairy cell differentiation, such as CD103, CD11c, CD123, and Cyclin D1, were also absent. The results support the diagnosis of a distinct B-cell lymphoid malignancy while effectively excluding other neoplastic conditions, including mantle cell lymphoma, chronic lymphocytic leukemia, and hairy cell leukemia.

Flow cytometric immunophenotyping demonstrated a clonal population of B cells with bright surface immunoglobulin and light chain restriction alongside strong expression of prototypical B-cell antigens (CD20, CD22, FMC7, CD79a). Notably, CD5 and CD23 expression were absent, and the cells did not express CD10, CD11c, CD103, or CD25, thereby effectively ruling out other common B-cell neoplasms such as CLL, hairy cell leukemia (HCL), or variants of low-grade lymphomas. In addition, the leukemic variant of mantle cell lymphoma (MCL) - commonly associated with the t(11;14)(q13;q32) translocation - was excluded by fluorescence in situ hybridization (FISH) analysis, which showed no evidence of this abnormality (Figure 3).

The patient was admitted for initiation of systemic therapy. Before proceeding with the R-B regimen, comprehensive laboratory evaluations were performed, including a comprehensive metabolic panel (CMP), coagulation panel, lactate dehydrogenase (LDH), arterial blood gas (ABG), and electrolytes, to identify any baseline abnormalities and establish the patient’s baseline values. A regimen of the R-B scheme was selected, consisting of rituximab (375 mg/m² on Day 1) and bendamustine (90 mg/m² on Days 1 and 2) every 28 days for up to 10 cycles.

**Figure 4:**
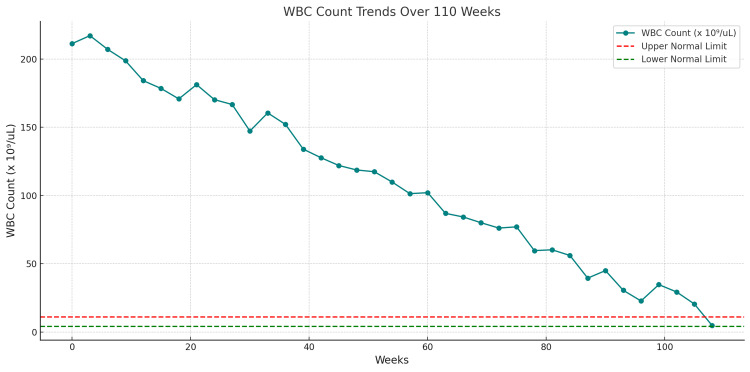
White blood cell (WBC) count trends over 110 weeks following treatment. This line chart demonstrates the trends in WBC count over a period of 110 weeks. The initial WBC count was markedly elevated at 213.82 × 10⁹/L, and a gradual reduction was observed, with fluctuations, until the final recorded count of 8.62 × 10⁹/L. The dashed red line represents the upper normal limit of the WBC count (11 × 10⁹/L), while the dashed green line indicates the lower normal limit (4 × 10⁹/L). These data reflect a positive response to the administered treatment, as the patient’s WBC count decreased progressively and approached normal levels over time. The fluctuations in WBC counts are consistent with treatment adjustments and disease dynamics but do not deviate from the overall downward trend.

Throughout treatment, the patient’s complete blood count (CBC) was closely monitored to assess hematologic response and therapy effectiveness. The trajectory of the patient’s WBC count over time, reflecting a positive response to therapy, is illustrated in Figure 4. During the chemotherapy, the patient showed no clinical or laboratory signs of tumor lysis syndrome (TLS), although prophylactic allopurinol was administered to mitigate the risk of TLS development. Additionally, the patient experienced no significant deterioration in their general health status and tolerated the treatment well without notable adverse events. 

In this patient, routine post-treatment laboratory evaluations were conducted to ensure the absence of residual disease and treatment-related complications. The CMP revealed normal levels of electrolytes, including sodium, potassium, chloride, and bicarbonate, as well as markers of renal function, such as blood urea nitrogen (BUN) and creatinine. Liver function tests, including alanine aminotransferase (ALT), aspartate aminotransferase (AST), alkaline phosphatase (ALP), and total bilirubin, were within normal ranges, indicating no hepatic dysfunction. LDH, a marker often associated with cellular turnover, was also normal, consistent with the absence of active disease. The coagulation panel, including prothrombin time (PT), international normalized ratio (INR), and partial thromboplastin time (PTT), demonstrated normal clotting function. These findings, together with the normal CBC, confirm the restoration of physiological balance and the patient’s excellent response to the treatment regimen, with no evidence of ongoing malignancy or systemic abnormalities (Table 2).

**Table 2:** Complete blood count (CBC) results after treatment in a patient with B-cell prolymphocytic leukemia. This table presents the patient’s CBC values after treatment, reflecting the normalization of several hematologic parameters compared to reference ranges. These results demonstrate hematologic recovery following therapy for B-cell prolymphocytic leukemia. Parameters such as white blood cell count (WBC), hemoglobin (HGB), and platelet levels have returned to within normal ranges, indicative of treatment efficacy and remission status.

Parameter	Patient value	Reference range
White blood cell count (WBC)	8.62 × 10⁹/L	4.0-11.0 × 10⁹/L
Red blood cell count (RBC)	4.62 × 10¹²/L	4.5-5.9 × 10¹²/L
Hemoglobin (HGB)	14.2 g/dL	13.5-17.5 g/dL (male)
Hematocrit (HCT)	42.5%	40%-52%
Mean corpuscular volume (MCV)	92.0 fL	80-100 fL
Mean corpuscular hemoglobin (MCH)	30.5 pg	27-31 pg
Mean corpuscular hemoglobin concentration (MCHC)	33.2%	32%-36%
Red cell distribution width (RDW-CV)	14.0%	11.5%-14.5%
Platelets (PLT)	225.4 × 10⁹/L	150-450 × 10⁹/L
Mean platelet volume (MPV)	8.29 fL	7.5-11.5 fL
Neutrophil count (NEUT#)	5.51 × 10⁹/L	1.8-7.8 x 10⁹/L
Neutrophil percentage (NEUT%)	63.89%	43.63%-76.6%
Lymphocyte count (LYMPH#)	2.26 × 10⁹/L	1.0-3 × 10⁹/L
Lymphocyte percentage (LYMPH%)	26.20%	16-43.5%
Monocyte count (MONO#)	0.60 × 10⁹/L	0.2-1.0 × 10⁹/L
Monocyte percentage (MONO%)	6.93%	4%-12.5%
Eosinophil count (EO#)	0.17 × 10⁹/L	0.0-0.5 × 10⁹/L
Eosinophil percentage (EO%)	1.97%	0.0-5%
Basophil count (BASO#)	0.09 × 10⁹/L	0-0.1 × 10⁹/L
Basophil percentage (BASO%)	1.00%	0-1%

The patient has since remained in clinical remission. Regular follow-ups, including comprehensive laboratory testing and imaging, showed no evidence of disease recurrence. As of October 2024, more than two years post-diagnosis, the patient remained asymptomatic with stable hematologic parameters and no signs of relapse.

To further confirm the remission status, minimal residual disease (MRD) analysis was conducted on a bone marrow sample collected on November 20, 2024. Immunophenotyping was performed using an eight-color flow cytometer (Agilent Advanteon Dx V0B5R3) with monoclonal antibodies targeting CD45, CD19, CD20, CD81, CD22, λ light chain, and κ light chain. The analysis registered over 2.5 million cells in duplicate samples, ensuring robust accuracy. The findings revealed no evidence of residual atypical cells associated with the primary disease. The B-cell lineage identified (3%) consisted of 19% mature polyclonal B lymphocytes and 79% hematogenous, indicative of normal bone marrow regeneration.

These results confirm the patient’s MRD-negative status, reinforcing the conclusion of complete remission and long-term disease-free survival. This case highlights the critical importance of advanced diagnostic tools and diligent post-treatment monitoring in achieving and verifying durable remission in aggressive hematologic malignancies like B-PLL.

Statistical analysis of the CBC trends revealed a significant reduction in the patient’s WBC count from an initial 213.82 × 10⁹/L to 8.62 × 10⁹/L posttreatment, illustrating a robust hematologic response to the R-B regimen (Figure 4). This decline followed a consistent downward trend (R² = 0.94 in regression analysis), underscoring the treatment's efficacy. Parallel improvements were observed in hemoglobin and platelet levels, with mean increases of 8.1 g/dL and 99 × 10⁹/L, respectively, throughout therapy.

Flow cytometric immunophenotyping documented an 85% clonal B-cell population expressing CD19/CD20 with light chain restriction at baseline, which diminished to undetectable levels post-treatment. This finding was accompanied by a concurrent rise in normal hematopoietic cell populations, indicative of bone marrow recovery.

All clinical and laboratory data were carefully logged in the institution's electronic medical records and subsequently analyzed using specialized statistical tools. This structured approach ensured precise tracking of the patient’s progress and reliable interpretation of treatment outcomes. These findings underscore the value of systematic data collection and analysis in managing rare and challenging conditions like B-PLL.

## Discussion

B-PLL is an exceedingly rare and aggressive mature B-cell malignancy, accounting for less than 1% of all lymphoid leukemias [2,3,5]. The median age at diagnosis is approximately 69 years, with a slight male predominance [5,6]. Clinical features often include marked lymphocytosis, splenomegaly, and constitutional *B* symptoms, while lymphadenopathy is less commonly observed [6]. Despite its distinct morphological and immunophenotypic features, B-PLL poses significant diagnostic and therapeutic challenges due to its aggressive clinical course and frequent resistance to conventional treatments [4,6,7].

Our patient, a 64-year-old male, presented with fever, night sweats, weight loss, and generalized lymphadenopathy, reflecting a typical constellation of symptoms associated with B-PLL [2,3,4]. His CBC at presentation revealed marked leukocytosis (213.82 × 10⁹/L) and anemia (HGB: 6.1 g/dL), consistent with reported cases [2,4,7]. Morphological examination of peripheral blood smear demonstrated prolymphocytes with a high nuclear-to-cytoplasmic ratio and prominent nucleoli. The bone marrow aspirate and flow cytometry findings heightened suspicion for the diagnosis by revealing a clonal B-cell population characterized by the expression of CD19, CD20, CD22, CD38, and CD45, along with λ light chain and IgM. Notably, the cells were negative for CD5, CD10, CD23, CD25, CD33, CD34, CD43, CD103, CD123, CD200, and CD11c. This immunophenotypic profile aligns with established diagnostic criteria for B-PLL, facilitating its distinction from CLL and other B-cell neoplasms [1,6,7]. Notably, distinguishing B-PLL from other B-cell neoplasms, such as CLL and MCL, can be challenging due to overlapping morphological and immunophenotypic features. However, the absence of the translocation t(11;14)(q13;q32), which is characteristic of MCL, serves as a critical distinguishing factor in confirming the diagnosis of B-PLL. This cytogenetic finding helps refine the diagnostic process and ensures accurate differentiation among these entities [1,7,8].

The literature underscores the pivotal role of TP53 mutations and deletions in driving the aggressive nature of B-PLL. Approximately 38% of cases exhibit these abnormalities, often correlating with chemoresistance and poor outcomes [8]. Other chromosomal abnormalities, such as deletions at 11q23 and 13q14, are also frequently implicated in B-PLL, suggesting a complex molecular landscape that influences disease behavior [8,9]. Recent studies have further elucidated the genetic underpinnings of B-PLL, highlighting the significance of MYC gene abnormalities and complex karyotypes in the disease's progression and prognosis [8]. Additionally, research has shown that patients with both MYC activation and 17p deletion, which includes the TP53 gene, have shorter overall survival and should be considered a high-risk *double-hit* subgroup [9,10].

Traditional chemotherapeutic regimens have limited efficacy in B-PLL, particularly in patients with adverse genetic profiles [11]. However, targeted therapies have emerged as promising options. Rituximab, a monoclonal antibody targeting CD20, combined with alkylating agents such as bendamustine, has shown clinical benefit in B-PLL. This approach was adopted in our patient, resulting in significant hematologic recovery and normalization of CBC values [4,6]. Posttreatment flow cytometry confirmed the absence of MRD-negative and revealed a predominance of polyclonal B-lymphocytes (19%) and B-lymphoid precursors (79%), consistent with remission [12]. Beyond traditional regimens, newer agents such as ibrutinib (a BTK inhibitor) and venetoclax (a BCL-2 inhibitor) have demonstrated efficacy in TP53-mutated cases, offering durable responses in some instances [13]. While these therapies were not utilized in our case, their growing role highlights the evolving landscape of B-PLL treatment.

Despite the availability of these therapeutic options, the prognosis for B-PLL remains guarded, with a median survival of less than three years [1,2,7,8]. Allogeneic stem cell transplantation offers a potential cure but is often limited by age and comorbidities [14]. Remarkably, despite its aggressive nature and poor prognosis, our patient achieved complete remission following the R-B regimen, with no evidence of disease recurrence over two years of follow-up. Our patient tolerated the treatment exceptionally well, with no significant adverse effects or complications reported during or after the therapy. Routine laboratory evaluations confirmed gradual improvement in hematologic parameters, and clinical monitoring indicated that he adapted well to the regimen without signs of TLS, organ dysfunction, or systemic complications.

After each cycle of R-B administration, the patient opted to return home under the care of his family, with a nurse available on-call 24/7 to address any potential concerns. This approach ensured continuity of care while allowing the patient to recover in a familiar environment, contributing positively to his quality of life during the treatment period. Given his excellent tolerance of therapy and the absence of significant functional impairments, oncology rehabilitation was not deemed necessary in this case.

Post-treatment flow cytometry confirmed MRD-negative status, with findings consistent with hematologic remission. This underscores the effectiveness of personalized treatment strategies in managing B-PLL and highlights the potential for durable outcomes even in high-risk patients.

## Conclusions

This case highlights the potential for favorable outcomes in B-PLL with timely and precise diagnosis combined with individualized treatment strategies. The significant remission achieved in our patient with an R-B regimen underscores the evolving importance of targeted therapies in managing this rare and aggressive malignancy. This report adds to the evidence supporting the use of R-B as a potential treatment option for patients with B-PLL, emphasizing the importance of comprehensive diagnostic evaluation and tailored therapeutic approaches in optimizing outcomes. However, the rarity of B-PLL poses challenges in establishing standardized treatment protocols, and the absence of long-term follow-up data beyond two years limits the ability to assess the durability of remission and late-onset adverse effects. Additionally, the lack of comparative studies on the efficacy of alternative regimens constrains our understanding of optimal treatment approaches. Continued research, including well-structured clinical trials, is essential to refine therapeutic strategies and improve prognostication for this challenging disease.
